# Economic potential and production determinants of selected neglected and conventional livestock species in Ogun and Oyo States, Nigeria: A comparative case study

**DOI:** 10.1016/j.vas.2026.100597

**Published:** 2026-02-13

**Authors:** Maria Oguche, Folasade O. Oke, Juliet Kariuki, Richard Oloo, Thomas Potthast, Mizeck G.G. Chagunda, Regina Birner

**Affiliations:** a Institute of Agricultural Science in the Tropics (Hans-Ruthenberg-Institute), University of Hohenheim, Germany; b Department of Agricultural Economics and Farm Management, Federal University of Agriculture, Abeokuta, Nigeria; c International Livestock Research Institute (ILRI), Kenya; d International Centre for Ethics in the Sciences and Humanities (IZEW), Eberhard Karls Universität of Tübingen, Germany; e Centre for Tropical Livestock Genetics and Health (CTLGH), Easter Bush Campus, University of Edinburgh, United Kingdom

**Keywords:** Nigeria, Neglected livestock species, Animal husbandry, Production system, Profitability, Resource efficiency

## Abstract

There are livestock species such as rabbit, guinea fowl, and grasscutter in Sub-Saharan Africa which are traditionally consumed but have not received as much attention and support from policy and research compared to conventional livestock species such as cattle, sheep, goat, pig and poultry. Despite the growing interest in such neglected livestock species (NLS), evidence on their integration, production, and drivers of adoption remain sparse. This comparative study investigates the production characteristics, profitability, and determinants of farmers’ choices between conventional and NLS production in Ogun and Oyo States, Nigeria. We conducted a quantitative survey of 183 smallholder livestock producers, selected using simple random and snowball sampling procedures across Ogun and Oyo States. Data were collected through questionnaires and analysed using descriptive statistics, logistic regression, and cost-benefit analysis. The findings show that women were disproportionately engaged in NLS production. Intensive and semi-intensive production systems were the most prevalent, driven by land scarcity and the adaptability of NLS to confined production environments. Feed emerged as the dominant cost component across both species, significantly impacting profitability. Logistic regression analysis showed that the choice of NLS production was positively and significantly associated with mode of land acquisition, profit, and access to credit. Profitability analysis revealed that NLS systems achieved a higher return on investment than conventional systems, offering a viable pathway for enhancing income, food security, and resilience among smallholders in the study areas. Conclusively, with an appropriate policy framework, neglected livestock species could contribute to a transformative shift in Southwestern Nigeria’s livestock landscape.

## Highlights

• Neglected livestock species (NLS) shows greater benefits than conventional.
• NLS offers potentials for improving smallholder livelihoods.
• Promoting NLS aids inclusive livestock transformation agenda.

## Graphical abstract

**Figure:**
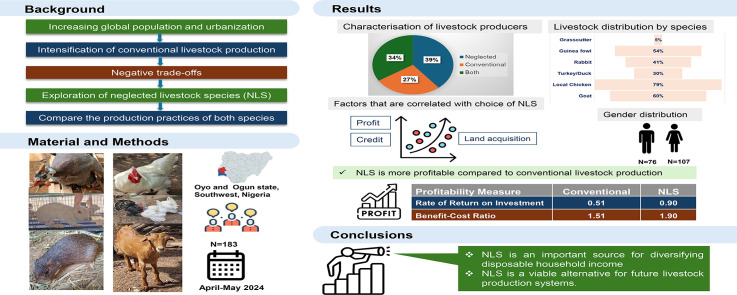

## 1 Introduction

The growth rate of human population in Sub-Saharan Africa was projected to rise from 2.4 % in 2023 to 3 % in 2024, with further increase to 4 % anticipated in 2025–2026 (World Bank Group, 2024). Nigeria, just as many other countries in Sub-Saharan Africa, is experiencing population increase that does not match its economic growth (Adeosun & Popogbe, 2021). The livestock sector in Nigeria plays a pivotal role in the economy – contributing about 20 % and 5.6 % to the agricultural and national Gross Domestic Product (GDP), respectively (Nigerian National Bureau of Statistics, 2025). The sector also contributes substantially to food security, income generation, and employment for millions of households – particularly in rural areas (Molina-Flores et al., 2020). This sector is mainly characterized by a diverse group of conventional livestock species, predominantly cattle, sheep, chicken, pig and goat (National Bureau of Statistics (NBS) and Federal Ministry of Agriculture and Food Security (FMAFS), 2024). However, there are other livestock species that also contribute to food security, nutrition, income generation and sustainable development but yet receive disproportionately limited attention, investment and prioritization within formal agricultural research, policy and development frameworks. A study by Oguche et al., 2024 terms such livestock species as “Neglected Livestock Species (NLS)”. Species considered to be neglected include rabbit, guinea fowl, grasscutter, and snail (Molina-flores et al., 2020; Oguche et al., 2024). In Nigeria, the production and management practices associated with livestock species at household level are reportedly influenced by several factors, including cultural preferences, market dynamics, and socio-economic conditions (Federal Ministry of Agriculture and Rural Development and World Bank Group (FMARD and WBG), 2020; Molina-Flores et al., 2020).

The categorisation of livestock species as conventional or neglected is essential for understanding the diverse socio-economic, cultural and ecological factors that influence how they are reared and the sustainability of the associated production practices. This involves understanding the various inputs, structures, practices, and dynamics of production which can considerably influence production outcomes.

While traditional farming has mostly relied on conventional livestock species, there is, however, a growing interest and investment in the production of NLS - leading to diversification in agricultural practices both regionally and nationally (Assan, 2014; Megersa et al., 2014). NLS are often perceived as more sustainable alternatives due to their lower resource requirements (Aluko & Salako, 2015; Yiva et al., 2014), comparatively quicker economic turnover (Ibitoye et al., 2019; Serem et al., 2013), and potential for enhanced profitability and empowerment (Adedeji & Osowe., 2015; Kale et al., 2016; Lammers et al., 2009). As such, understanding the production dynamics and strategies employed by farmers raising these species is important for their accelerated adoption and integration into current production systems.

Despite their importance, existing literature on livestock production in Southwest Nigeria primarily focuses on conventional livestock species, leaving a notable gap in our understanding of NLS. It has been suggested that socio-economic factors investment capabilities, and access to markets are key determinants in production practices, ultimately influencing farmers' choices (Sodjinou et al., 2015; Stringer et al., 2020; Tatis Diaz et al., 2022). Moreover, significant variables that influence the decision-making processes of livestock farmers include income level, education, gender, access to credit, market access, and social networks (Naicker et al., 2025; Olumba et al., 2025; Shitaye et al., 2024; Stringer et al., 2020). Despite these previous reports, studies that integrate socio-economic determinants with the typology and characterisation of livestock production are scarce. More specifically, there also is a considerable gap in the existing literature regarding the typology, characterisation, and socio-economic factors that influence both conventional and neglected livestock species production systems in Southwest Nigeria. Consequently, using an exploratory field survey spanning two demographically distinct States in Southwest Nigeria, this study aimed to: a) characterise the socio-economic traits of producers and their associated production practices; b) examine socio-economic factors that drive farmers’ choices of livestock production; and c) assess the profitability of neglected livestock species in comparison to conventional livestock species. The findings of this study are expected to inform policies and practices that will enhance the livestock sector in the region and beyond.

### 1.1 Theoretical and conceptual frameworks

Considering the novelty of comparison between conventional and neglected livestock species production practices, this study adopts the term “neglected livestock species” and draws insights from the conceptual framework by Oguche et al., 2024. The reconceptualisation of livestock species outside the conventional cattle-sheep-chicken-goat-pig taxonomy - which have been described as “micro-livestock” (National Research Council, 1991), “minilivestock” (Hardouin, 1995), “minor species” (USFDA, 2025) by Oguche et al., 2024 - to NLS presents a paradigm shift that highlights institutional and structural dimensions. In global health, the World Health Organization’s classification of Neglected Tropical Diseases (NTDs) are characterised by their systematic exclusion from research, development funding and public health prioritization (WHO, 2011). Similarly, “Neglected and Underutilized Species (NUS)” is used to describe crops that despite their positive contribution to food security, receive limited research attention and policy support compared to major staple crops (FAO, 2017; Padulosi et al., 2013). These characterisations mirror the institutional status of the neglected species of focus: grasscutter, rabbit and guinea fowl which are not necessarily absent from agricultural systems but are underrepresented in breeding programs, value chain development initiatives and policy frameworks.

In Nigeria, official statistics for livestock often refer more to conventional livestock and rarely NLS. For example, the National Agricultural Sample survey 2022/2023 by the Nigerian National Bureau of Statistics reports cattle, goats, sheep, pigs, and chicken as main livestock raised with grasscutter, and rabbit absent from these official statistics (National Bureau of Statistics (NBS) and Federal Ministry of Agriculture and Food Security (FMAFS), 2024). NLS were also found to be excluded from the Agricultural Promotion Policy of 2016–2020 (Federal Ministry of Agriculture & Rural Development, 2016).

Subsequent to the classification of both categories of livestock, it can be assumed that the neglect of these species in comparison to the conventional ones could also directly affect the livelihoods of their producers. Literature reports that NLS species producers lack access to formulated feeds, lack veterinary services due to lack of practitioners in these species, and lack of credit (Akinola et al., 2015; Anang et al., 2011; Owen & Dike, 2012). From the socio-economic perspective, the neglect could explain why the value chains of these species remain informal, fragmented with limited value addition, lack of processing and storage infrastructures (Adedeji & Osowe, 2015; Akinola et al., 2015; Owen & Dike, 2012; Ume & Ezeano, 2017). The comparative advantages of NLS characteristics over conventional species such as their nutritious protein sources (Adedeji & Osowe, 2015; Akinola et al., 2015), higher economic returns (Adedeji & Osowe, 2015; Benjamin et al., 2009; Ume & Ezeano, 2017), efficient feed conversion and prolificacy (Avornyo et al., 2016; Kale et al., 2016), adaptability to smallholder farming systems with land constraints (Aluko & Salako, 2015; Opara, 2010), entry point for women’s empowerment (Adedeji & Osowe, 2015; Oseni & Lukefahr, 2014) and cultural uses (Akinola et al., 2015; Aluko & Salako, 2015; Ebegbulem, 2018) creates pathways for increased institutional inclusion.

Using Ogun and Oyo States in the Southwestern region of Nigeria as case study, this study further leverages on the Farm Household Production Theory and Sustainable Livelihood Framework to explore livestock production choices and outcomes among smallholder farmers under the influence of socio-economic, institutional, and resource-related factors.

Farm Household Production Theory posits farm households as entities that production and consumption decisions are simultaneously subject to certain constraints like land, labour, capital and technology (Singh et al., 1986). Unlike profit-maximizing firms, farm households aim to maximize utility, which includes income, food security, and risk reduction. In livestock production, households choose production systems and species based on expected returns, labour requirements, household food needs, and exposure to risk. Since resources such as land and capital are limited, households may prefer production systems that offer lower costs of production and faster turnover. In the context of this study, neglected livestock species, such as rabbits, guinea fowl, grasscutters, and other small livestock are assumed to be rational production choices for households facing land scarcity, capital constraints, and livelihood risk. These species often require less land, lower initial investment, and shorter production cycles compared to conventional livestock such as cattle and large ruminants. This theory provides the background for analysing how household characteristics such as farm size, income sources, household size, and access to productive resources influence the choice between conventional livestock and NLS production.

The Sustainable Livelihoods Framework (Department for International Development (DFID), 1999) provides a comprehensive approach in understanding how households pool assets to pursue livelihood strategies under different scenarios of vulnerability. The framework identifies five types of capital assets: human (education, age), natural (land, feed), physical (housing, equipment), financial (savings, income), and social capital (associations, networks). The relevance of this theory to this study is explained by the fact that livestock production, particularly, embracing NLS serves as a livelihood strategy that enhances resilience by reducing income variability, improving food availability, and spreading risk. In locations plagued with land pressure, climate variability, and market uncertainty, NLS production can improve returns per unit of land and contribute to livelihood sustainability. In this study, the Sustainable Livelihoods Framework supports the analysis of how access to assets and institutional support influences livestock production strategies and how these strategies affect income and other livelihood outcomes.

**Fig. 1:**
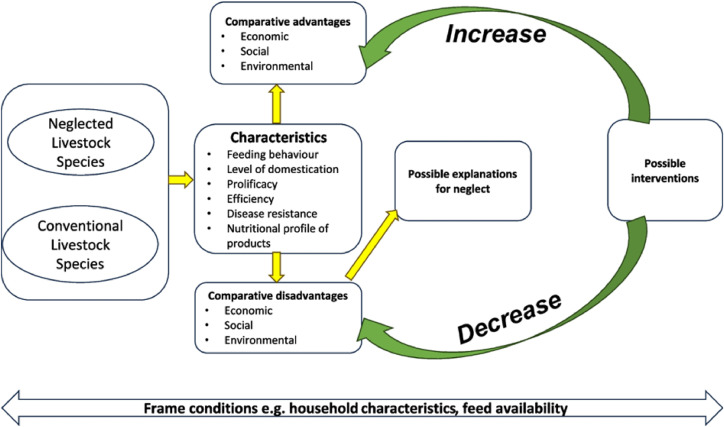
Conceptual framework adapted from Oguche et al. (2024).

Drawing from these integrated theoretical perspectives, this study conceptualizes (Fig. 1) and tests that frame conditions such as household characteristics (e.g., education, household size, gender, income sources; resource constraints in form of land, and capital availability), institutional factors (extension access, cooperative membership) as well as the biological features of the livestock species to create profitability, jointly influences the choice of livestock species among households (conventional versus neglected).

## 2 Methodology

### 2.1 Study area

**Fig. 2:**
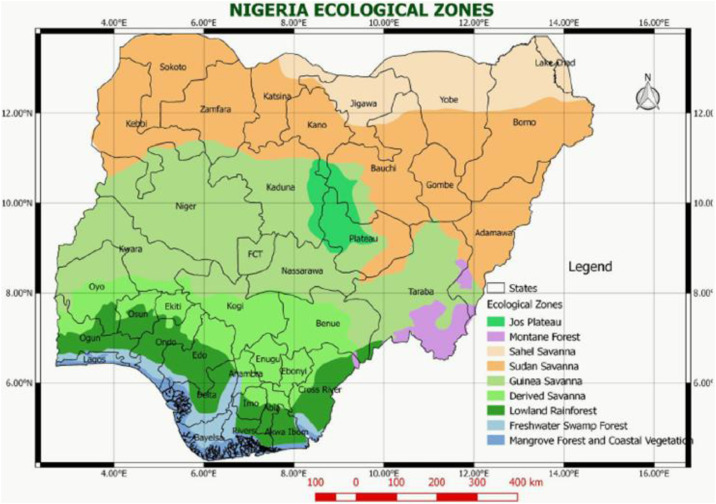
Map of Nigeria showing agroecological zones (Source: FORMECU., (1998) and FAO (2020)).

The study was carried out in Ogun and Oyo states of the Southwestern region of Nigeria (Fig. 2). The states are within the derived savannah and lowland rainforest agroecological zones of Nigeria (Fig. 2).

**Fig. 3:**
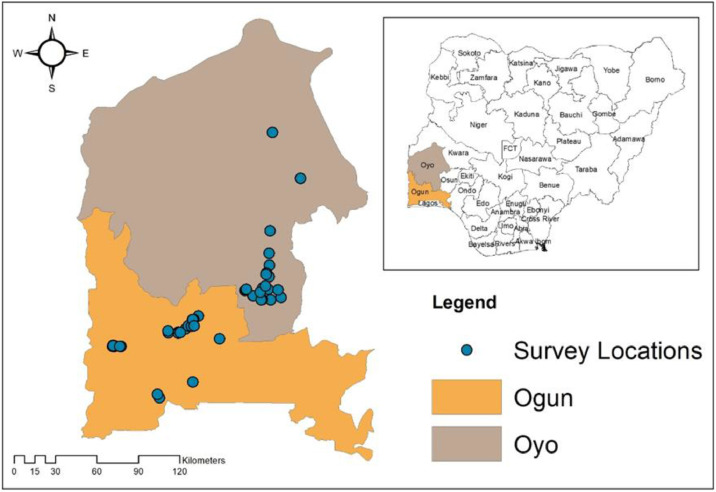
Map of Nigeria map showing the study area and the locations of surveyed farmers.

The states are a conducive environment to produce a variety of livestock species due to the presence of diverse forage species suitable for livestock feed.(Agbo et al., 2023; Federal Fertilizer Department, Federal Ministry of Agriculture & Rural Development, 2011; Fischer et al., 2021; Food and Agriculture Organization of the United Nations (FAO) and the International Institute for Applied System Analysis (IIASA), 2021). Four-fifths of the respondents were from Ogun State, whereas one-fifth were from Oyo State. Around 20 % of Ogun State’s total area is forest reserve which is reported to be suitable for livestock production (Obayelu et al., 2015) – and is divided into three senatorial^1^ districts: Ogun Central, Ogun East and Ogun West. Oyo state is also divided into 3 senatorial districts: Oyo Central, Oyo North, and Oyo South. These districts served as the primary study locations, and they offered a diverse range of socio-economic, cultural, and infrastructural characteristics, which are critical in understanding the focus of the study (Fig. 3).

### 2.2 Ethical considerations of research

The study did not involve the collection of clinical data. With regard to interviews, verbal informed consent was obtained from all participants prior to their involvement in the study and also regarding further research usage of the results of this study. To ensure confidentiality, all data was anonymized, and responses were securely stored. The study received approval from the ethics committee of the University of Hohenheim (Ref. No. 2024/07_Oguche).

### 2.3 Study design and data collection

The study was conducted in April - May 2024 as an exploratory case study applying a quantitative method approach in data collection. Employing an exploratory approach was necessitated due to the absence of baseline data on the prevalence and distribution (Stebbins, 2001) of the production of neglected livestock species in the study area. The comparative framework of the study required sampling households producing conventional, neglected or both species. Therefore, the species of interest for both groups are: conventional – Turkey, duck, local chicken, goat; and neglected livestock species - grasscutter, guinea fowl, rabbit. Households keeping only conventional species serve as the comparison group not experiencing institutional exclusion, while those keeping only neglected species experience institutional exclusion and the mixed-category households enable within-household comparative analysis controlling for household fixed effects.

The study initiated data collection through partnership with the Federal University of Agriculture, Abeokuta (FUNAAB), which provided a list of livestock farmers in Ogun State compiled through the university’s Agricultural Media Resources and Extension Centre (AMREC)^2^. This list provided an entry point into the livestock farming community. Individuals involved in the production or rearing of at least one of the species of focus, were eligible for inclusion and had a chance of being selected. The list was screened through conducted telephone calls to identify farmers keeping the livestock species category of focus. This process generated the initial respondents in Ogun state, which were mainly the conventional species producers (*n* = 38 and *n* = 19 NLS producers), revealing the inadequacy of existing lists for neglected species producers.

This led to the adjustment of the sampling strategy and Snowball sampling (Biernacki & Waldorf, 1981; Noy, 2008) was subsequently employed. In this approach, the initial respondents (first contact farmer) recommended other individuals engaged in similar livestock production activities. This process simultaneously identified other conventional species producers as some neglected species producers also keep conventional livestock. This technique proved effective in fostering trust between the researchers and respondents, as some respondents preferred to be contacted through a fellow farmer from their local area or cooperative societies. Snowball sampling continued until theoretical saturation (Glaser & Strauss, 1999) indicators emerged such as new referrals increasingly identified already interviewed farmers.

During fieldwork in Ogun state communities along the Ogun-Oyo boundary, we came across farmers who referenced production activities in adjacent Oyo state communities. This observation, mixed with theoretical interest in comparing production practices across a more populated state, motivated a purposive geographical expansion to Oyo state. In Oyo state, the study relied entirely on snowball sampling initiated through referrals from Ogun state respondents.

The surveys in both states were conducted in English and Yoruba, the local language of the region. A total of 183 respondents, representing targeted livestock producers directly managing the household’s production activities were interviewed. The study’s sampling did not employ stratified sampling by district with predetermined quotas; rather, respondents were distributed across 6 different senatorial districts which emerged from the adaptive sampling process. The respondents as shown in Table 1 came from Ogun Central, Ogun East, Ogun West, Oyo Central, Oyo North, and Oyo South senatorial districts with Oyo North having the least (5) and Ogun West the most (87) respondents.

**Table 1:** Distribution of respondents based on senatorial districts.

State	Senatorial districts	N (183)
Ogun state (*N* = 145)	Ogun Central	36
	Ogun East	22
	Ogun West	87
Oyo state (*N* = 38)	Oyo Central	11
	Oyo North	5
	Oyo South	22

Cumulatively, Ogun and Oyo States had 79 % and 21 % share of total respondents, respectively. The resulting 145:38 ratio between Ogun and Oyo respondents are due to: initial focus on Ogun state as primary case study site; b) later timing of Oyo expansion; and c) resource constraints limiting extensive Oyo state field work.

Quantitative data from these respondents were collected using questionnaires administered using the KoboToolbox (Das, 2024), an open-source platform, established in 2005 by Phuong Pham and Patrick Vinck (Pham & Vinck, 2005). Data were analysed using descriptive and inferential statistics using STATA as well as Microsoft Excel.

### 2.4 Data analysis

#### 2.4.1 Logistic regression analysis

The socio-economic factors influencing the choice of livestock species category (conventional versus neglected) among the producers were analysed. The dependent variable (Y) in this binary choice model is dichotomous, taking a value of 1 if the producer chose neglected livestock production and 0 if otherwise. Thus, we use a non-linear logistic regression framework which can be denoted as follows:

(1) $$ln\left(P_{i}/\left(1-P_{i}\right)\right)=\beta_{o}+\sum_{j=1}^{k}\beta_{j}\;X_{ij}+\varepsilon_{i}$$

Where $P_{i}$ is the probability that respondent engages in neglected livestock species production, $\beta_{o}$ is the model intercept, $\beta_{j}$ are the model pararameters, $\;X_{ij}$ are the explanatory variables and $\varepsilon_{i}$ is the residual error term.

#### 2.4.2 Profitability analysis

To determine the profit level per production cycle, costs incurred and returns from conventional and neglected livestock species farming enterprise were estimated separately including the cost of all inputs used (fixed and variable), and the quantity of output (animals). Since the study was conducted in Nigeria, costs estimates were collected in the local currency, the Nigerian Naira - NGN (₦). These costs were subsequently converted to United States dollars ($) using the Central Bank of Nigeria’s (CBN) reference average exchange rates for the Naira for the month of April and May 2024 - =₦1335.28/US$ (average exchange rate of the naira per US dollar in April 2024=₦1235.80/US$; May 2024=₦1434.75/US$)(Central Bank of Nigeria 2024).

Profitability ratios like Net Farm Income (NFI), Gross Margin (GM), Rate of Return on investment (RORI) and Benefit-Cost Ratio (BCR) were calculated from the cost and return analysis. This can be expressed mathematically below:

(2) NetFarmIncome(NFI)=Profit(π)=TR−TC

(3) GrossMargin(GM)=TR−TVC

(4) Benefit−CostRatio(BCR)=TR/TC

(5) RateofReturnonInvestment(ROI)=NFI/TC

Where:

Total cost (TC) = Total Fixed Cost (TFC) + Total Variable Cost (TVC)
TR = Total Revenue ($) = Output (Q) × Price (P) = PQ
TVC = Total Variable Cost ($)
TFC = Total Fixed Cost ($)
*P*= Unit price of output ($)
*Q*= Livestock total output (number)

According to Oke et al. (2022) an agricultural enterprise is profitable if Total Revenue (TR) is greater than Total Cost (TC), the Benefit-Cost ratio (BCR) value exceeds 1, the Rate of Return on Investment (ROI) value is above zero and gross margin and Net Farm Income (profit) values are positive.

## 3 Results

### 3.1 Socio-economic characteristics of livestock producers

**Fig. 4:**
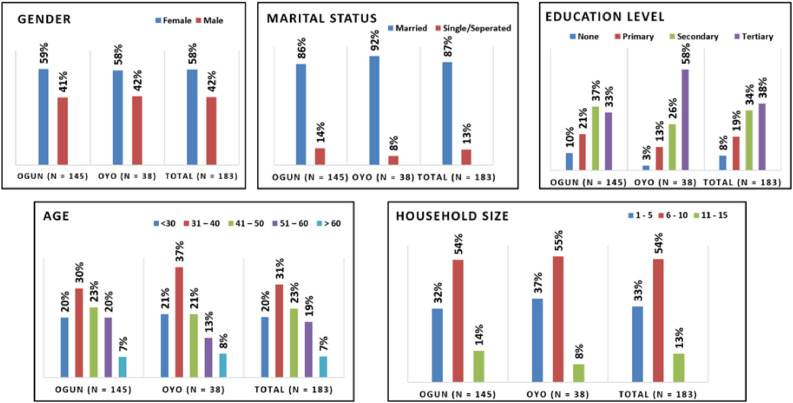
Socio-economic characteristics of the interviewed livestock producers.

It was found that diverse demographics were involved in the production of conventional and neglected livestock species (Fig. 4). More females (58 %) participated in the study and majority of the respondents were married (87 %). Overall, almost all respondents (92 %) had basic education (primary education). However, further examination revealed that 34 % had secondary education, while 38 % had tertiary education. Notably, majority of the respondents were aged below 60 years and the age group of 31–40 represented the largest portion. Household size distribution showed that most respondents (54 %) reported to have come from a household comprising between 6–10 persons while 33 % lived in households consisting of 1–5 persons.

### 3.2 Types of livestock producers in the study area

**Fig. 5:**
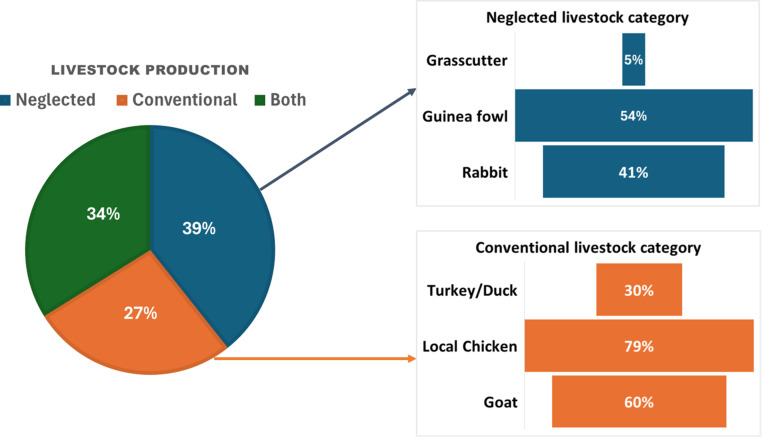
Classification of conventional and NLS species type, owned by respondents in the study (Source: Field survey, 2024.). *Percentages were derived based on the assumption that respondents could have multiple responses. Respondents were banded into two broad categories (conventional and neglected), with respondents having the option to choose more than one response within and across categories. Values have been rounded to their nearest whole numbers.*

The respondents in this study kept a diverse range of the species of focus for both conventional and NLS categories (Table 2). In our results, duck and turkey were grouped together because the farmers treated them as one group due to their similarities in production practices, and the derived products, i.e. meat and eggs. Our results show that 39 % of the respondents kept only NLS, 27 % kept only conventional species and 34 % kept both. The predominant neglected species was guinea fowl (54 %) and predominant conventional species was local chicken (79 %). Grasscutter (5 %) and Turkey/duck (30 %) were the least represented among the neglected and conventional livestock categories, respectively (Fig. 5).

**Table 2:** Classification of conventional and NLS species type, owned by respondents in the study.

Variables	Ogun % (*N*= 145)	Oyo % (*N*= 38)	Pooled % (*N*= 183)
**Livestock production**			
Neglected	37 % (54)	47 % (18)	39 % (72)
Conventional	26 % (38)	29 % (11)	27 % (49)
Both	37 % (53)	24 % (9)	34 % (62)
**Neglected**^a^	**Ogun**% (***N***= **107**)	**Oyo% (*N*=27)**	**Pooled% (*N*= 134)**
Grasscutter	4 % (4)	11 % (3)	5 % (7)
Guinea fowl	64 % (68)	15 % (4)	54 % (72)
Rabbit	33 % (35)	74 % (20)	41 % (55)
**Conventional**^a^	**Ogun% (*N*=91)**	**Oyo% (*N*=20)**	**Pooled% (*N*= 111)**
Turkey/Duck	32 % (29)	20 % (4)	30 % (33)
Local Chicken	87 % (79)	45 % (9)	79 % (88)
Goat	62 % (56)	55 % (11)	60 % (67)

a *Percentages were derived based on the assumption that respondents could have multiple responses. Respondents were banded into two broad categories (conventional and neglected), with respondents having the option to choose more than one response within and across categories. Values have been rounded to their nearest whole numbers.*

### 3.3 Production systems practiced by respondents per species

The survey showed that three different production systems can be distinguished in the study area, which can be characterized as (1) extensive/free range, (2) semi-intensive, and (3) intensive (Table 3). The intensive system, which involves controlled feeding and husbandry practices was most frequently practiced overall (44 %), followed by the semi-intensive system (37 %) and the extensive system (15 %). However, there was considerable variation between livestock species. Regarding conventional livestock, the extensive system was most common for local chicken (56 %) and goats (39 %). Regarding NLS, guinea fowl were the most frequently raised in such systems (42 %). In Table 3, no farmer uses extensive system for grasscutter, and only 1 % indicating practicing semi-intensive system, often citing concerns over disappearance of this livestock species. Goats were most frequently raised in semi-intensive systems. Further details can be derived from Table 3.

**Table 3:** Production systems practiced by respondents by species.

Livestock Production System	States					
	Ogun State (*n* = 145)		Oyo State (*n* = 38)		Pooled (*n* = 183)	
	Frequency	%	Frequency	%	Frequency	%
Extensive/free range	26	18	1	3	27	15
Semi-intensive	55	38	12	32	67	37
Intensive*	56	39	24	63	80	44
Mixed	8	6	1	3	9	5
**Extensive/Free range system**						
Grasscutter	**-**	**-**	**-**	**-**	**-**	**-**
Guinea fowl*	15	44	-	-	15	42
Rabbit	4	12	-	-	4	11
Turkey/Duck	5	15	-	-	5	14
Local chicken*	17	50	3	150	20	56
Goat	11	32	3	150	14	39
**Semi-intensive System**						
Grasscutter	1	2	-	-	1	1
Guinea fowl*	23	37	1	8	24	36
Rabbit	2	3	7	54	9	13
Turkey/Duck	10	16	2	15	12	18
Local chicken*	32	51	2	15	34	51
Goat	28	44	3	23	31	46
**Intensive System**						
Grasscutter	3	5	2	8	5	6
Guinea fowl	22	34	2	8	24	27
Rabbit*	25	39	18	72	43	48
Turkey/duck	13	20	1	4	14	16
Local chicken*	20	31	6	24	26	29
Goat	13	20	5	20	18	20

Source: Field survey, 2024. *Percentages were derived based on the assumption that respondents could have multiple responses. Respondents were banded into three broad categories (extensive, semi-intensive and intensive), with respondents having the option to choose more than one response within and across categories. Values have been rounded to their nearest whole numbers.*

NOTE:.

⁎ implies responses with the highest frequencies for each category of livestock and production systems.

### 3.4 Resource availability associated with conventional and neglected livestock species

#### 3.4.1 Feeding

**Fig. 6:**
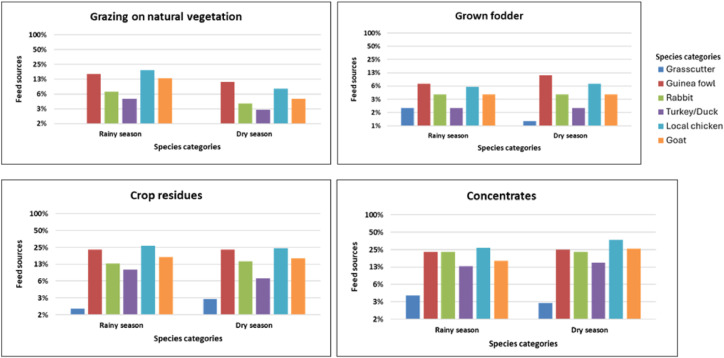
Feed sources by species during the rainy and dry seasons (Source: Field survey, 2024).

Table 4 summarizes the frequency and seasonal variation of different feed types and sources, showing how practices differ based on seasonality and availability. 56 % of feed resources in livestock production are locally produced, while 25 % is imported from outside the region, and the remainder comes from both sources. Concentrate feed was a dominant component of the nutritional base in both dry and rainy seasons. Crop residues have gained importance as a feed source for about 51 % of the farmers, with no significant seasonal differences in the frequencies. Farmers indicated as a reason for the high use of concentrates to be the limited land available for grazing or to grow own fodder. To ensure adequate nutrition, farmers reported that they practice feeding their livestock with mixed feeds from different sources, such as supplementing forage with concentrates. Fig. 6 shows further distribution of feed sources by species during the rainy and dry seasons.

**Table 4:** Feed and feeding materials.

Feed types	States				Pooled (*n* = 183)	
	Ogun State (*n* = 145)		Oyo State (*n* = 38)			
	Frequency	%	Frequency	%	Frequency	%
Locally produced feed	91	63 %	11	29 %	102	56 %
Imported	29	20 %	17	45 %	46	25 %
Both	25	17 %	10	26 %	35	19 %
**Feed sources**^a^						
Grazing on natural vegetation	49	34 %	8	21 %	57	31 %
Grown fodder	33	23 %	6	16 %	39	21 %
Crop residue	75	52 %	18	47 %	93	51 %
Concentrates	94	65 %	33	87 %	127	69 %

NOTE: * implies responses with the highest frequencies for each category of livestock and feed sources.

a *Percentages were derived based on the assumption that respondents could have multiple responses and values have been rounded to their nearest whole numbers.*

#### 3.4.2 Access to land and other resources

Access to land, a vital element in livestock production, was found to vary in both availability and mode of acquisition. The pattern for acquisition shows that private purchase (51 % of the respondents) was the dominant way of acquiring ownership of land while acquisition through inheritance was reported by 29 % (Table 5). In terms of farm size, a clear relationship between scale of operation and resource availability was observed. The majority of the farmers surveyed were smallholders managing <3 hectares (ha) of farmland. A large majority of the interviewed farmers (72 %) had access to technical support from extension agents. Credit and financial facilities were not accessible to 61 % of the livestock producers. Cooperative membership was popular, as 83 % of our respondents were members of cooperatives. Such group memberships were reported to come with benefits, such as enhanced access to support structures like extension and credits.

**Table 5:** Access to land and other resources.

Variables	States						Pooled (*n* = 183) % (N)
	Ogun State (*n* = 145)			Oyo State (*n* = 38)			
	% (N) Conventional	% (N) NLS	% (N) Both	% (N) Conventional	% (N) NLS	% (N) Both	
**Mode of land acquisition**	*Percentages were derived based on the assumption that respondents could have multiple responses. Values have been rounded to their nearest whole numbers.*						
Private purchase	11 % (16)	19 % (28)	21 % (31)	18 % (7)	26 % (10)	5 % (2)	51 % (94)
Inheritance	10 % (14)	13 % (19)	8 % (12)	-	13 % (5)	8 % (3)	29 % (53)
Communal purchase	-	1 % (1)	1 % (1)	3 % (1)	-	-	2 % (3)
Government owned	-	1 % (1)	-	-	3 % (1)	-	1 % (2)
Rental	6 % (8)	9 % (13)	4 % (6)	11 % (4)	26 % (10)	-	22 % (41)
Others: Backyard of residential house	1 % (1)	3 % (5)	-	-	3 % (1)	5 % (2)	5 % (9)
**Farm size (ha)**							
< 3	19 % (27)	28 % (40)	25 % (36)	29 % (11)	34 % (13)	18 % (7)	73 % (134)
3 −6	6 % (9)	6 % (9)	6 % (9)	-	8 % (3)	3 % (1)	17 % (31)
>6	1 % (2)	3 % (5)	6 % (8)	-	8 % (3)	-%	10 % (18)
**Access to extension services**							
Yes	18 % (26)	20 % (29)	35 % (51)	24 % (9)	39 % (15)	5 % (2)	72 % (132)
No	8 % (12)	17 % (25)	1 % (2)	5 % (2)	11 % (4)	16 % (6)	28 % (51)
**Access to credit**							
Yes	9 % (13)	12 % (18)	20 % (29)	5 % (2)	8 % (3)	16 % (6)	39 % (71)
No	17 % (25)	25 % (36)	17 % (24)	24 % (9)	42 % (16)	5 % (2)	61 % (112)
**Membership of cooperatives**							
Yes	24 % (35)	33 % (48)	30 % (44)	16 % (6)	34 % (13)	16 % (6)	83 % (152)
No	2 % (3)	4 % (6)	6 % (9)	13 % (5)	16 % (6)	5 % (2)	17 % (31)

Source: Field Survey, 2024.

### 3.5 Factors that influence choice of NLS versus conventional species

The coefficient of factors that influence farmers’ choice to keep NLS or conventional species has been presented in Table 6 and their marginal effects in Table 7. The observed differences in significance levels of the variables between the coefficients and the marginal effects is attributed to small sample size in this study. Level of education (coefficient = −0.575, *p*= 0.007), mode of land acquisition (coefficient = 0.681, *p*= 0.005), profit (coefficient =1.46e-06, *p*= 0.000), and access to credit (coefficient =1.201, *p*= 0.004), significantly influenced the choice of livestock species produced in the study area. Higher educational levels decreased the likelihood of keeping NLS, possibly because better educated farmers with higher human capital (education, training) may prefer conventional species due to better institutional support and formal market access.

**Table 6:** Logistic regression results of factors that influence choice of NLS versus conventional species.

Variables	Coefficient	Standard Error	z	p-value
Age	−0.01	0.015	0.64	0.519
Gender	0.269	0.399	0.68	0.500
Marital Status	0.098	0.070	0.66	0.511
Household Size	−0.046	0.067	−0.49	0.621
Level of Education	−0.575***	0.214	2.69	0.007
Other income sources	−0.334	0.426	0.79	0.432
Farm Size	0.062	0.090	0.69	0.489
Mode of land acquisition	0.681***	0.243	2.80	0.005
Profit	1.46e-06***	3.22e-07	4.52	0.000
Cooperative membership	−0.268	0.503	0.53	0.593
Access to credit	1.201***	0.420	2.86	0.004
Constant	−0.973***	1.217	0.80	0.424

Pseudo r-squared 0.197.

Chi-square 45.144.

Akaike crit. (AIC) 207.552.

Bayesian crit. (BIC) 245.53.

Prob > chi2 0.000.

Log likelihood −91.776.

LR chi2(11) 45.14.

Number of observations 175.

***, **, * means significant at 1 %, 5 % and 10 % respectively.

Source: Field Survey Data Analysis, 2024.

**Table 7:** Marginal effects of the logistics estimates of the factors that influence choice of NLS versus conventional species.

Variables	dy/dx	Standard Error	z	p-value
Age	−0.002	0.003	0.518	0.007
Gender	0.047	0.070	0.497	0.089
Marital Status	0.017	0.110	0.875	0.198
Household Size	−0.008	0.012	0.509	0.032
Level of Education	−0.101***	0.035	2.89	0.004
Other income Source	−0.059	0.074	0.429	0.204
Farm Size	0.011	0.016	0.690	0.020
Mode of land acquisition	0.120***	0.039	3.05	0.043
Profit from livestock production	0.000***	0.000	5.89	0.000
Cooperative membership	−0.047	0.088	0.002	0.078
Access to credit	0.211***	0.068	3.11	0.002

(*) dy/dx is for discrete change from 0 – 1 in the case of dummy variables and for a marginal change evaluated at mean values in case of continuous variables.

***, **, * means significant at 1 %, 5 % and 10 % respectively.

Source: Field Survey Data Analysis, 2024.

Mode of land acquisition was a significant predictor of the type of livestock species kept (*P* < 0.05). Farmers with own land either purchased privately or inherited have a higher likelihood of engaging in NLS than those with land acquired through communal purchase, government owned or rental. The ability of a farmer to have own land would increase the likelihood of being involved in the neglected livestock production system by 12 % probably. The perceived profit potential expectedly influenced species selection; it was positively and significantly correlated with NLS (*P* < 0.01). The respondents who have access to credit had a higher likelihood of keeping NLS than their counterparts (*p* < 0.05). Having access to credit increased the likelihood of producing NLS by 21 %.

### 3.6 Economic analysis of conventional and NLS production

The result of the economic analysis (costs, return and profitability) of conventional livestock and neglected livestock production monthly is presented in Table 8. There was no major difference in the share of variable cost in the total cost of production (95 % among conventional producers and 98 % among the neglected producers). Accordingly, the share of fixed costs (depreciation value), accounting for 4.86 % among the conventional livestock producers and 1.54 % for the neglected livestock producers. The cost of feed accounted for under two-fifths and slightly less than half of the total expenditure incurred in conventional and neglected livestock production, respectively. The mean values of the total variables and fixed costs were only marginally higher for NLS compared to the conventional livestock production. In also comparing the mean values of total revenue and net farm income, the values for NLS were considerably greater than for the conventional livestock species production. This implies that neglected livestock species production was more profitable in our sample than the conventional livestock species production. The gross margin for keeping conventional species was $22.47 and for NLS it was $34.16, which further indicates that farmers who kept NLS made more profit relative to the variable inputs used than farmers who kept conventional livestock species.

**Table 8:** Cost and return analysis (in USD - $) of conventional and NLS production.

S/N	Items	Mean Amount (Conventional)	Mean Amount (NLS)	%TC (Conventional)	%TC (NLS)
1A	**Total Revenue (sale of livestock)**	**60.51**	**71.02**		
2	**Variable cost**				
i.	Stocking	3.61	2.64	9.03	7.05
ii.	Feed	15.35	16.89	38.39	45.13
iii.	Labour	4.91	1.25	12.29	3.33
iv.	Stables repair/maintenance	3.65	3.48	9.12	9.30
v.	Feed additives	1.71	2.61	4.28	6.98
vi.	Veterinary	3.08	2.81	7.69	7.51
vii.	Fuel/Energy	1.69	3.04	4.24	8.13
viii.	Transport	4.03	4.13	10.09	11.02
B	**Total Variable Cost (TVC)**	**38.04**	**36.86**		
C	**Gross Margin (GM) = TR-TVC**	22.47	34.16		
3	**Fixed Cost (Depreciated)**				
i	Land	0.36	0.09	0.89	0.25
ii	Feeder & Drinker	0.03	0.03	0.07	0.08
iv	Housing	1.56	0.45	3.91	1.21
D	**Total Fixed Cost (TFC)**	**1.95**	**0.57**		
E	**Total Cost (TC) = TFC+TVC**	39.99	37.43		
F	**Profit/Net Farm Income (NFI) =GM-TFC**	20.52	33.58		
G	**Rate of Return on Investment (RORI) = NFI/TC**	**0.51**	**0.90**		
H	**Benefit-Cost Ratio (BCR) = TR/TC**	**1.51**	**1.90**		

Computed from field survey, 2024.

Values from Naira to USD were converted at the rates of $1 = ₦1335.28.

The rate of return on investment (RORI), as an indicator of profitability, was 0.51 and 0.90 for the keeping conventional species versus NLS, respectively. This implies that for every $1.00 invested in the conventional and neglected livestock production systems, $0.51 and $0.90 were obtained as revenue, respectively. These results clearly indicate the comparative potential of the neglected livestock production in the study area.

Despite this, the statistical analysis of the differences in profit using Welch’s *t*-test revealed that the difference was not significant (*p*> 0.05). The mean profit for NLS was significantly higher than that for conventional livestock species (Table 9) by at least 15 USD.

**Table 9:** Welch’s *t*-test comparing mean profit between NLS and conventional livestock species showing estimated means, p-value and confidence interval.

T-value	Degree of freedom	P-value	Mean (Conventional)	Mean (NLS)	95 % confidence interval of difference (USD)
−0.905	108.371	0.36722263	20.5245	33.58386	–41.64648 to –15.52776

## 4 Discussion

This study analysed the socio-economic characteristics of producers and management systems associated with farmers keeping conventional versus neglected livestock species in Ogun and Oyo state, Nigeria. This study also examined factors that influence farmer’s choice of livestock species and assessed the profitability of the different livestock species production in the study area.

### 4.1 Socio-economic traits of producers

We set out to identify the socio-economic traits of producers in our study area. Respondents in the household studied were generally below 60 years of age – with a substantial proportion being educated. Respondents were mostly women (∼58 %).

Our findings highlight the gendered nature of livestock production practices – as majority of the respondents were women. This observation could be attributed to the livestock species under consideration. Generally, NLS are easier to handle and require less capital (Aluko & Salako, 2015; Ebegbulem, 2018; Niba et al., 2012; Owen & Dike, 2012; Yiva et al., 2014), aligning with the prevailing socio-economic dynamics in the study area – where women have less capital compared to men (Adeosun & Owolabi, 2021; Jaiyeola & Adeyeye, 2021; Odozi & Odozi, 2012). Thus, it could be argued that the availability of capital is one of the drivers underlying the decision by women to go into the production of NLS. Indeed, men have been reported to be predominantly involved in production systems involving larger livestock such as cattle while the women mostly manage smaller livestock (Galiè et al., 2019; IFAD, 2010). As it relates to the socio-economic disparity between men and women in the study area within the broader context of Nigeria (Adeosun & Owolabi, 2021; Odozi & Odozi, 2012), men are likely to take on capital intensive ventures such as keeping larger livestock compared to the women folks. Notwithstanding, more men rather than women have been reported to be more involved in rabbit production in Ghana – highlighting the nuanced nature of findings in this regard. This seeming contradiction may be influenced by geographical and socio-cultural differences. For example, Bonis-Profumo et al. (2022) found that in Timor-Leste, women required the consent of their husbands before producing, selling, or purchasing livestock. This reveals that the involvement of women in NLS may largely be influenced by socio-cultural circumstances. Thus, there is a need to situate the interpretation of the findings of similar studies in a societal context, noting that findings may be constrained.

Notably, despite respondents, mostly young people, having their main jobs, they were involved in the production of NLS. This could be a strategy for earning additional disposable household income, considering that salaries from main jobs may not be sufficient. Our observation of the age distribution (predominantly above 30 years) aligns with the findings of Mutua et al. (2017) and Verbeek et al. (2007) who reported similar age brackets in small ruminant production in Kenya. In recent years there has been a strong push for young people to engage in agriculture, including small ruminant production – as it offers a pathway for achieving food security (Mensah et al., 2018; Nchanji et al., 2023; Yeboah et al., 2020).

Interestingly, we found that ∼72 % of respondents cumulatively had secondary and tertiary education – in agreement with recent surge of educated individuals in agriculture including NLS production (Chah & Ndofor-Foleng, 2013; Koné et al., 2018; Mensah et al., 2024). It has been argued that educated individuals are comparatively likely to record higher production output due to the likelihood of adopting technology and keeping adequate records (Oli et al., 2025; Quddus, 2022). Taken together, our findings that women are more involved in the production of NLS, provides a pathway for empowering women economically in the study area – while exploring ways to replicate same in other areas.

### 4.2 Characterisation of production practices and system of management

In our sampled population, NLS-only producers were 39 % and conventional species-only producers were 27 % – indicating an increasing recognition on the importance of NLS in households. Studies have emphasised that NLS are particularly significant for smallholder farmers, where they often serve as source of income, provision of diverse nutritional products including eggs, meat, and their adaptability to adverse rearing conditions (Ibitoye et al., 2019; Kale et al., 2016; Molina-flores et al., 2020; Moreki & Seabo, 2012).

Although the NLS-only producers were more, farmers involved in an integrated system of keeping conventional and neglected livestock were also substantially present. A previous report found that integrated practices such as small ruminant rearing and poultry farming are prevalent in our study area - with farmers focusing on traits and systems that enhance productivity and sustainability (Oyekunle & Lawal-Adebowale, 2021). This practice is reported to serve as a risk mitigation mechanism for farmers as it provides multiples sources of income (Mosnier et al., 2022). Further, it could be assumed that this system enables resource-use efficiency by utilising feed wastages, fibrous forages, milled meals, kitchen wastes and other by-products which can be shared across both groups. Also, in terms of land/space requirement, keeping both categories of conventional and neglected serves as a strategic utilization of available land space which is often limited (van Zanten et al., 2016). While challenges such as logistical constraints as well as technical know-how could negatively affect farmers (Abdul-Majid et al., 2024; Smidt & Jokonya, 2022), integrated practices may have an overall more beneficial outcome.

Livestock husbandry and feeding in the study areas were predominantly intensive with both conventional and neglected livestock relying on feed concentrates. The prevalence of this production system could be attributed to the land availability characteristic of the study area, since a previous study in Burkina Faso, Mali and Nigeria reported that most livestock keepers in areas with land-use change from agricultural to non-agricultural activities are characterised by intensive production systems (Amadou et al., 2012). In this regard, intensive and semi-intensive systems are emerging to address land-use conflicts and increasing productivity by optimising production (Amadou et al., 2012; Gérard, 2020; Herrero et al., 2009; Simpkin et al., 2020). Notably, availability of land for production continues to dwindle in settings such as our study areas, mainly due to competing land-use interests (Aliyu & Amadu, 2017; Korah & Wimberly, 2024). Without any intervention, this situation may imperil livestock production and put food secutiy at risk.

Consequently, this situation has transformed traditional livestock-keeping practices by risking the welfare of animals, as the drive to increase production within limited space raises ethical issues about how these adaptations affect the optimal growth and functionality of the animals, given their biology. For instance, grasscutter are reported to thrive in dense grasses close to rivers and swamps, and herbaceous vegetation where they can have good cover (Aluko & Salako, 2015). Yet, our results (Table 3), indicate that grasscutter were generally being kept under intensive system, denying them of the conditions that help them to thrive. Taken together, these issues bring to the fore a need for a holistic approach in the formulation of policies on animal husbandry – also systematically considering animal welfare.

Our study also found that concentrates were the dominant feed source. This likely reflects an additional advantage of intensive system, where farmers rely more on concentrated feeds to optimise feed utilisation using nutrient-dense rations. Some are commercially formulated for use across different livestock species, as producers increasingly rely on common base ingredients (maize, soybean meal, wheat offal) adjusted to meet the nutritional need of each species to promote growth and production. However, the reliance on intensive systems and concentrated feed also brings the concern regarding some negative trade-offs with regards to sustainability, animal welfare, increased production costs and vulnerability to feed market volatility. Animals in confined conditions are prone to the risks of spreading pathogenic viruses, sharing diffused antibiotic-resistant bacteria and physical cruelty (Anomaly, 2014). For example, the 2020 case of Rabbit Hemorrhagic Disease (RHD) outbreak in Nigeria (Oyewo et al., 2022) with rabbitries operating semi-intensive and intensive management systems (usually placed on formulated feeds) recording the highest mortality (Daodu et al., 2023).

Altogether, it is important for future studies to examine causal relationship between urbanisation and the type of production systems practiced. While it was observed that intensive system was predominated, the lack of information on why that was the case presents a new avenue for further enquiry. Nonetheless, the increasing land-use conflicts call for a re-evaluation of production systems to conform to the current realties, while balancing that adequately against ethical concerns.

### 4.3 Nexus between demographic variables, their influence on farmer’s choice of livestock species and the resulting profitability

Our study further sought to understand how socio-economic factors shape farmers' choices of livestock species while also assessing which of the compared livestock species categories (conventional and neglected livestock) offers greater profitability. Results showed that factors such as level of education, mode of land acquisition, and access to credit significantly influence choice of livestock species (conventional vs NLS) adopted.

The negative relationship between education level and probability of choosing NLS represents one of the study’s theoretical findings. Higher educational attainment significantly influences conventional species adoption, implying more educated farmers would not choose NLS despite their perceived advantages but leverage their human capital more in conventional livestock species production. This could be due to the availability of complementary institutional supports such as extension services, research-generated information, formal value chains and credit access.

The common mode of land acquisition was found to be through private purchase. This method of acquisition indicates that limited space may be available for grazing or growing fodder on communal land, which adds financial pressure on producers. The high cost of land acquisition in Nigeria (Federal Ministry of Agriculture & Rural Development & World Bank Group, 2020) could naturally encourage producers’ choice of adopting intensive systems - as grazing becomes economically unfeasible in urban areas (Olumba et al., 2019). This spatial challenge reinforces intensive production practices such as the use of cages to optimise production within confined spaces. Such practices not only address the limitations of land but also allows for more efficient management through tighter control of husbandry practices. This, in turn, enhances overall health and productivity, driving economic benefit for farmers. The positive relationship between mode of land and NLS production (coefficient = 0.681, *p*= 0.005) further portrays private land ownership as a need for intensive system of production documented in the findings. The prevalence of cage-based systems for the NLS requires infrastructure investment (housing, fencing, water systems) that rational farmers will only undertake with secure tenure.

This may also further reflect the differential land requirements across species. NLS, in comparison to conventional species, require less land (Aluko & Salako, 2015; Opara, 2010; Owen & Dike, 2012). However, some studies have reported that, when there is a marginal increase in farm size, there is an increased preference for conventional livestock species, compared to NLS (Dibissa & Peters, 1999; National Sample Survey Organisation Ministry of Statistics & Programme Implementation Government of India, 2006; Manyike et al., 2025). This is because conventional livestocks, comparatively, require a larger space to meet their welfare needs, including grazing and movements (Barry, 2021; Henn et al., 2024; Siegford et al., 2008). In general, the choice of production system, among other factors, is influenced by access to land and land size (Kolade & Harpham, 2014). Thus, our findings provide additional evidence of how choice of livestock species produced is influenced by availability of land.

It was also found that profit maximisation and economic viability influenced the production decisions made as perceived higher profitability incentivizes investment into specific systems. Moreover, diversification of income sources also affected the choice of neglected livestock production significantly as having multiple streams of income increases producers' household flexibility in investing in livestock production systems. These findings suggest that profitability is an important driver in the choice of livestock species. Relatedly, Duluins et al. (2022), reported that economic benefits encourage livestock production system transitions. Indeed, it was found that NLS was more profitable than conventional species production, based on the RORI and BCR rations reported by Zorn et al. (2018). Studies have also reported a significant difference at the marketing price of the various products ranging from eggs to meat. Balma and Richard (2016) report a substantially higher profitability ratio in Ghana for the sale of meat and eggs from guinea fowl compared to beef, chicken, pork, or mutton. This is also similar to other studies conducted in Nigeria by Benjamin et al. (2009), Ume and Ezeano (2017), and Adedeji and Osowe (2015), who reported a higher price for the sale of NLS and their products compared to their conventional counterparts. There appear to be a growing consensus that NLS is more profitable than conventional species based on evidence from this study and others. However, studies available are too scant to support a general conclusion, especially considering variability across the different socio-economic contexts in which these studies are conducted.

Overall, we found that NLS play a complementary role to conventional livestock species and provide additional disposable income to households as well as support the attainment of food security. Under conditions of uncertainty faced by smallholder farmers, our findings offer additional evidence supporting the rationale and benefits of both integrated and diversified livestock production practices. Nonetheless, the growing adoption of some intensive production system practices such as the usage of cages, confinement, and high reliance on feed concentrates raise animal welfare concerns. Further research that analyses the entire value chain of NLS to identify the trade-offs that exist for and between humans, animals, and the environment is recommended to enhance our understanding of how these interlinked aspects interact.

Furthermore, recent policy developments – inclusion of “micro-livestock” in the Nigeria Livestock Roadmap for Productivity Improvement and Resilience 2020–2026 and the establishment of a dedicated Federal Ministry of Livestock Development (FMLD) in July 2025 – create an evolving institutional landscape that this study could not fully anticipate. However, this emerging policy recognition acknowledges the institutional gaps this study empirically documents at the household level and reinforces the findings of this study, particularly the use of the the terminology "neglect".

## 5 Conclusion

This study aimed to characterise the socio-economic traits of producers and their associated production practices, examine the socio-economic factors that influence farmers’ choices of livestock production, and assess the profitability of neglected livestock species in comparison with conventional livestock species. Our findings provide insights into the typology, characterisation and socio-economic determinants of conventional and neglected livestock species production in Ogun and Oyo States, Nigeria. Integrated theoretical framework synthesizing Farm Household Production Theory, the Sustainable Livelihoods Framework, and Oguche et al.’s (2024) conceptualisation of neglected livestock species, were employed. This study’s methodology focused on household assets, household-level production decisions, species characteristics and observable institutional access (extension, credit and cooperatives). The practice of neglected livestock species production in intensive and semi-intensive system was common among smallholder farmers in the study areas.

This study highlights that species choice and production outcomes emerge from three-way interactions: household assets, species characteristics and institutional contexts. It can be argued that while farmers often maintain and utilize a diverse range of species valued for different reasons – policies, regulations, subsidies and development programs should not focus on only one dimension (e.g., credit access without addressing extension capacity) or a narrower and limited set of commercially significant or politically visible species. It is therefore recommended that future research focuses on optimisation of mixed species systems, particularly involving labor-efficient management practices and nutritional complementarity between species. This would be valuable within the context of current climate crisis and market volatility, justifying a greater institutional support for producers pursuing a diversification strategy. There is a need for balanced approach and policy interventions that consider environmental, economic, and animal welfare dimensions of livestock production tailored to specific system, without undermining the economic interests of producers.

### 5.1 Limitations of study

This study faces some limitations, including small sample size and external validity. We want to acknowledge that we do not have a completely random sample and the sample size is not large enough to be statistically representative. We have also not used statistical techniques that account for sample selection bias. However, in spite of these limitations, we see the value of the study in providing first-hand empirical data on the economics of NLS.

## Data availability

The data that support the findings of this study were not deposited in an official repository and are available from the corresponding author upon request.

## Ethical statement

The study did not involve the collection of clinical data. The study received approval from the ethics committee of the University of Hohenheim (Ref. No. 2024/07_Oguche).

## CRediT authorship contribution statement

**Maria Oguche:** Writing – review & editing, Writing – original draft, Visualization, Software, Project administration, Methodology, Investigation, Formal analysis, Data curation, Conceptualization. **Folasade O. Oke:** Writing – review & editing, Software, Methodology, Formal analysis, Data curation. **Juliet Kariuki:** Writing – review & editing, Project administration, Methodology, Conceptualization. **Richard Oloo:** Writing – review & editing, Software, Methodology, Formal analysis. **Thomas Potthast:** Writing – review & editing, Validation, Supervision, Resources, Project administration, Methodology, Funding acquisition, Data curation, Conceptualization. **Mizeck G.G. Chagunda:** Writing – review & editing, Validation, Supervision, Resources, Project administration, Methodology, Funding acquisition, Data curation, Conceptualization. **Regina Birner:** Writing – review & editing, Validation, Supervision, Resources, Project administration, Methodology, Funding acquisition, Data curation, Conceptualization.

## Declaration of competing interest

The authors declare that they have no known competing financial interests or personal relationships that could have appeared to influence the work reported in this paper.

## Figures and Tables

**Annex 1:** Feed types and sources for Fig. 6.

Feed types	States				Pooled (*n* = 183)	
	Ogun State (*n* = 145)		Oyo State (*n* = 38)			
	Frequency %		Frequency %		Frequency %	
Grazing on natural vegetation	Rainy season	Dry season	Rainy season	Dry season	Rainy season	Dry season
Grasscutter	**-**	**-**	**-**	**-**	**-**	**-**
Guinea fowl*	19 %	14 %	5 %	-	16 %	11 %
Rabbit	6 %	4 %	13 %	-	7 %	4 %
Turkey/Duck	6 %	3 %	3 %	5 %	5 %	3 %
Local chicken*	21 %	10 %	11 %	-	19 %	8 %
Goat	14 %	6 %	5 %	-	13 %	5 %
**Grown fodder**						
Grasscutter	1 %	1 %	3 %	3 %	2 %	1 %
Guinea fowl*	9 %	14 %	-	-	7 %	11 %
Rabbit	3 %	4 %	8 %	3 %	4 %	4 %
Turkey/Duck	2 %	3 %	-	0 %	2 %	2 %
Local chicken*	7 %	8 %	3 %	0 %	6 %	7 %
Goat	4 %	4 %	5 %	3 %	4 %	4 %
**Crop residue**						
Grasscutter	1 %	2 %	3 %	5 %	2 %	3 %
Guinea fowl*	28 %	26 %	5 %	11 %	23 %	23 %
Rabbit	10 %	12 %	26 %	24 %	13 %	14 %
Turkey/duck	10 %	9 %	8 %	0 %	10 %	7 %
Local chicken*	29 %	30 %	18 %	0 %	27 %	24 %
Goat	18 %	19 %	16 %	5 %	17 %	16 %
**Concentrates**						
Grasscutter	2 %	1 %	11 %	11 %	4 %	3 %
Guinea fowl*	27 %	29 %	8 %	11 %	23 %	25 %
Rabbit	15 %	15 %	53 %	55 %	23 %	23 %
Turkey/duck	14 %	16 %	11 %	13 %	13 %	15 %
Local chicken*	30 %	37 %	18 %	34 %	27 %	37 %
Goat	14 %	25 %	21 %	29 %	16 %	26 %

NOTE:.

⁎ implies responses with the highest frequencies for each category of livestock and feed sources.

## Annex

Annex 1

## Footnotes

1 This is the delineation used in Nigeria including some West African countries as against provinces in European countries. All the 20 LGAs in the states were compressed into 3 senatorial districts.

2 Agricultural Media Resources and Extension Center (AMREC) of the Federal University of Agriculture, Abeokuta (FUNAAB) is responsible for all the extension/community engagement of the University.
